# Effects of Weight-Related Self-Stigma and Smartphone Addiction on Female University Students’ Physical Activity Levels

**DOI:** 10.3390/ijerph19052631

**Published:** 2022-02-24

**Authors:** Mohsen Saffari, Jung-Sheng Chen, Hung-Ching Wu, Xavier C. C. Fung, Chih-Cheng Chang, Yen-Ling Chang, Ruckwongpatr Kamolthip, Marc N. Potenza, I-Ching Lin, Chung-Ying Lin

**Affiliations:** 1 Health Research Center, Life Style Institute, Baqiyatallah University of Medical Sciences, Tehran 1435916471, Iran; m.saffari@bmsu.ac.ir; 2 Health Education Department, Faculty of Health, Baqiyatallah University of Medical Sciences, Tehran 1435916471, Iran; 3 Department of Medical Research, E-Da Hospital, Kaohsiung 82445, Taiwan; ed113187@edah.org.tw; 4 Department of Public Health, College of Medicine, National Cheng Kung University, Tainan 70101, Taiwan; sb7091094@gs.ncku.edu.tw; 5 Department of Social Worker, Chi Mei Medical Center, Liouying, Tainan 73657, Taiwan; 6 Department of Rehabilitation Sciences, Faculty of Health and Social Sciences, The Hong Kong Polytechnic University, Hung Hom, Hong Kong, China; xavier-cc.fung@connect.polyu.hk; 7 Department of Psychiatry, Chi Mei Medical Center, Tainan 70246, Taiwan; rabiata@mail.chimei.org.tw; 8 Department of Health Psychology, Chang Jung Christian University, Tainan 71101, Taiwan; 9 Department of Family Medicine, Cardinal Tien Hospital, New Taipei 23445, Taiwan; th.yenlingchang@gmail.com; 10 Institute of Allied Health Sciences, College of Medicine, National Cheng Kung University, Tainan 70101, Taiwan; ta8097034@ncku.edu.tw; 11 Departments of Psychiatry and Neuroscience and the Child Study Center, School of Medicine, Yale University, New Haven, CT 06511, USA; marc.potenza@yale.edu; 12 Connecticut Council on Problem Gambling, Wethersfield, CT 06109, USA; 13 Connecticut Mental Health Center, New Haven, CT 06519, USA; 14 Wu Tsai Institute, Yale University, New Haven, CT 06510, USA; 15 Department of Healthcare Administration, Asia University, Taichung 41354, Taiwan; 16 Department of Family Medicine, Asia University Hospital, Taichung 41354, Taiwan; 17 Department of Kinesiology, Health, and Leisure, Chienkuo Technology University, Changhua 50094, Taiwan; 18 Biostatistics Consulting Center, National Cheng Kung University Hospital, College of Medicine, National Cheng Kung University, Tainan 70101, Taiwan; 19 Department of Occupational Therapy, College of Medicine, National Cheng Kung University, Tainan 70101, Taiwan

**Keywords:** female, physical activity, smartphone use, weight stigma, young adults, addictive behaviors, internet addiction disorder

## Abstract

Physical inactivity is a common health problem in female college students, and factors such as weight self-stigma and smartphone addiction may negatively impact physical activity in this population. The aim of the current study was to investigate the associations between these variables and identify the moderating effects of smartphone addiction between weight stigma and physical activity. Using a cross-sectional study, information on the level of physical activity in the past week, weight-related self-stigma, and smartphone addiction, as well as demographics, were collected via an online survey from 391 female college students in Taiwan. Participants in two groups of moderate to high and low physical activity were compared using a zero-order bivariate correlation in terms of independent variables. A moderated mediation model using Model 14 in the Hayes’ PROCESS macro with 1000 bootstrapping resamples was applied to assess moderation effects. There were significant differences in terms of weight status, smartphone addiction, and weight stigma between active and inactive groups (*p* < 0.001). All independent variables except for age were positively correlated (0.14 < r < 0.45). Multivariate regression models indicated that weight status was associated with weight stigma (odds ratio [OR] = 9.13, *p* < 0.001; 95% CI = 6.90, 11.35). Weight status (OR = 0.47, *p* = 0.03; 95% CI = 0.23, 0.93), weight stigma (OR = 0.96, *p* = 0.03; 95% CI = 0.922, 0.997), and smartphone addiction (OR = 0.11, *p* = 0.003; 95% CI = 0.03, 0.47) were associated with physical activity. The moderating role of smartphone addiction on the association between weight stigma and physical activity was also identified (OR = 1.05, *p* = 0.049; 95% CI = 1.0001, 1.1004). The moderating effect of smartphone addiction on the association between weight stigma and physical activity suggests that designing interventions to address the negative impacts of both weight stigma and smartphone addiction may help to improve physical activity in female college students.

## 1. Introduction

Physical inactivity is a leading risk factor for non-communicable diseases such as diabetes, cardiovascular diseases, and mental health problems such as anxiety and depression [1,2]. It has been reported that physical inactivity accounts for nearly 6% of the world’s deaths [3]. In contrast, physical activity (PA), especially for young people such as college students, has many beneficial effects [4]. The movement from high school to a new environment such as college/university may be a challenging stage in human development. Such transitions may be associated with mood disorders, psychological distress, and a decreased self-concept because this transition often requires students to adapt and address new issues, including exposure to a novel space and interactions with people from different cultures and values [5,6,7].

Students usually attempt to become accepted by their peer groups, and thus having good body fitness and maintaining attractiveness, particularly for female students, is often a considerable concern [8]. Therefore, engaging in PA often helps more than improving just one’s psychological state and prevent problems (e.g., distress and depression); that is, PA engagement may contribute to better adaptation with the transition to the new college/university environment and its requirements as well as improving fitness and cognitive functions for students [9,10]. Therefore, according to the World Health Organization, adults should have a weekly program of PA including at least 150 min of moderate aerobic activity or a half time of that (75 min) of vigorous PA [11].

The phenomenon of overweight/obesity is now an epidemic not only in developed countries but also in many developing Asian countries, with overweight/obesity estimated to be impacting more than 600 million people worldwide [12,13]. Lifestyle changes including the increase of sedentary behaviors along with a decrease of PA have been linked to overweight/obesity, especially among young women [9]. A considerable proportion of college students experience weight gain during college [8]. One study found that up through the second year of university, students gained 2.7 kg on average due to reduced PA and increased time on the internet and spent studying [14]. Similarly, Gropper et al. [15] found an average weight gain of 3.0 kg over the college period [15]. Moreover, a high percentage of university students in the United States and other countries do not meet PA and dietary recommendations of a healthy lifestyle [16,17]. Wang [18] found that, in a sample of 4000 college students in China, more than 75 percent did not follow the recommended amounts of PA [18]. The situation is worse among female college students, and they have less intention and lower levels of PA engagement than males, while they are more vulnerable to health problems and likely experience problems related to physical inactivity, about 20% more than males [19,20].

Body mass index has been inversely associated with health status, especially aspects of psychological health such as self-esteem and self-concept [21,22]. Sociocultural beliefs toward body shape may expose people, particularly individuals with overweight/obesity, to weight teasing and shame regarding weight [23]. This phenomenon is widespread in young people and college students, often due to peer pressure, and may generate considerable distress leading to poor health outcomes [24].

The prevalence of weight stigma is increasing in both developed and developing areas, and approximately 20–35% of youth have experienced weight-related stigma [25]. Internal negative beliefs regarding body weight (internalized weight stigma) and teasing about weight (as an external source of weight stigma) have been associated with poorer mental well-being and may lead to problems such as depression, anxiety, low self-esteem, eating disorders, and weight gain and avoidance of PA [26,27]. Unfortunately, some healthcare providers may perceive individuals with overweight/obesity as lazy, weak-willed, or undisciplined, with little or no intention or motivation to get treatment [28]. These assumptions additionally may prevent such people to make lifestyle changes or receive healthcare due to social stigmatization. Self-stigmatization along with social teasing regarding weight may produce negative, self-deprecating beliefs that may worsen an individual’s general health [21]. Experiencing self-stigma regarding weight may reduce successful behaviors to lose weight and may discourage people from exercising [29]. Meanwhile, it may encourage people toward sedentary behaviors such as internet surfing, TV watching, online gaming, and excessive use of mobile phones [29,30].

Smartphone use may negatively impact physical and mental health. Excessive mobile phone use has been termed problematic mobile phone use, cell phone overuse, smartphone addiction, or dependence, and is typically considered a behavioral addiction [31]. Smartphone addiction (SPA) may be common among young adults, in particular college students, in part because of interests to develop social relationships in virtual networks, the existence of several online platforms to make such relationships, and skills in using modern technologies via their phones [31,32]. SPA has been associated with poor well-being, especially psychological (e.g., poor sleep quality, feelings of loneliness, mental fatigue, role conflict, and depression) [31,33]. SPA may also lead to somatic problems such as headache, painful neck and wrist, and visual impairment [34].

For college students, other negative outcomes including poor academic achievement and problems with interpersonal communications have also been linked to SPA [5]. Furthermore, the prevalence of SPA in women is greater than men, and young people between the ages of 18 and 22 are the largest growing population of smartphone users, suggesting that female versus male college students may be at higher risk of SPA [35].

Although smartphones may be used to encourage people to engage in PA, SPA may directly or indirectly decrease PA in college students [34]. SPA may increase the likelihood of sedentary behaviors and consequently reduce the quality or quantity of moderate to vigorous PA [36]. PA may be a significant negative predictor of SPA among university students [37]. SPA may be closely associated with low degrees of motivation for doing PA and actual engagement [31]. Together, these findings suggest that although there are likely relationships between weight-related self-stigma (WRSS) and PA in female college students, SPA may influence this relationship, although this possibility has not been directly examined. Therefore, the possible moderating effects of SPA on this proposed relationship should be directly examined to find better solutions to increase PA in female college students.

**Figure 1:**
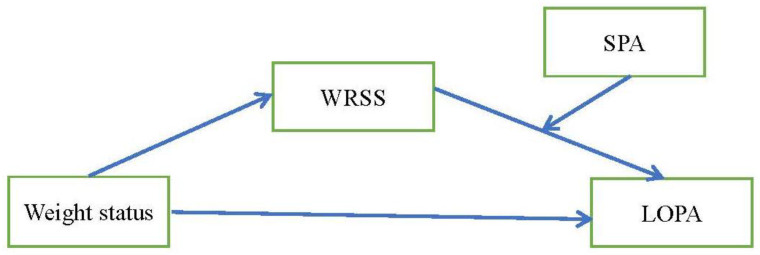
The hypothesized moderated meditation model to explain the moderator role of smartphone addiction (SPA) between the association of weight-related self-stigma (WRSS) and level of physical activity (LOPA), and the mediator role of WRSS between the association of weight status and LOPA in female college students.

Thus, the aim of this study was to use a moderated mediation model to examine the moderator role of SPA (i.e., if SPA moderated the association between WRSS and level of PA) and the mediator role of WRSS (i.e., if WRSS mediated the association between weight status and level of PA) in a sample of Taiwanese female college students. We hypothesized that there would be a negative relationship between WRSS and PA and that SPA would moderate this relationship; moreover, WRSS would be a significant mediator in the association between weight status and level of PA (Figure 1).

## 2. Materials and Methods

### 2.1. Participants and Data Collection

This study was approved by the institutional review board of Chi Mei Medical Center (IRB approval number: 11007-006). Google Forms was the platform used for data collection for this online survey. The survey was distributed using a hyperlink via different social networking methods (e.g., LINE and emails). The online survey included several questionnaires (please see the Measures section below for details), demographic information, and electronic informed consent. The research purpose, eligibility for participation, and rights of withdrawal were clearly presented on the first page of the Google Forms. After participants accepted informed consent, they could continue the survey.

Inclusion criteria were (1) female and over 20 years old, (2) a current university student, (3) having the ability to understand the Chinese questionnaire and the meaning of the questions, and (4) having a cellphone to connect with the internet in the past week. The survey period for recruiting participants was between August and September 2021. During this period, we sent out 800 invitations and received 600 responses (response rate = 75.0%). Among the 600 responses, 209 were invalid because the respondents were males and did not fit with the inclusion criteria. Therefore, we had 391 eligible participants for data analysis. The retained 391 eligible participants have completed all the online questionnaires without missing because all the survey items were set to be compulsory.

### 2.2. Measures

#### 2.2.1. Physical Activity: International Physical Activity Questionnaire-Short Form (IPAQ-SF)

The IPAQ-SF measures the level of PA in the past week [38]. There are seven items querying time spent engaging in different levels of PA. A sample item is, “During the last 7 days, on how many days did you do vigorous physical activities?” After each item, respondents are requested to provide the time spent (minutes) on that level of PA. Then, the metabolic equivalent of task (MET) was calculated according to different levels of physical activities (MET = 1 for sitting; 3.3 for walking; 4 for moderate physical activities; 8 for vigorous physical activities). Although levels of PA should be in a range (i.e., light PA has 1 to 3 MET, moderate PA has 3 to 6 MET, and vigorous PA has more than 6 MET), the range of MET was not used in the IPAQ for easy calculation. For example, if an individual spent (1) two hours on three days running, which is vigorous, (2) one hour on two days jogging, which is moderate, and (3) 30 min walking on seven days, the MET for this individual is: (8 × 120 min × 3 days) + (4 × 60 min × 2 days) + (3.3 × 30 min × 7 days) = 4053. With the use of the IPAQ, participants were classified into a group with a moderate to a high level of PA if (1) they had engaged in vigorous-intensity activities at least 20 min per day for 3 or more days; (2) they had engaged in moderate-intensity activities or walking at least 30 min per day for 5 or more days; or (3) they had a total of 600 or more MET-minutes per week. The IPAQ has demonstrated satisfactory test-retest reliability (intraclass correlation coefficient = 0.79) [39]. The present study used the Chinese version of IPAQ-SF.

#### 2.2.2. Weight-Related Self-Stigma: Weight Self-Stigma Questionnaire (WSSQ)

The WSSQ, a 12-item questionnaire that measures WRSS [40], consists of two distinct subscales that assess self-devaluation and fear of enacted stigma due to one’s body weight, respectively (six items for each subscale). The participants were instructed to respond on a 5-point Likert-type scale, from 1 (strongly disagree) to 5 (strongly agree). A higher score in the WSSQ indicates a higher level of weight-related self-stigma. Moreover, a total score of the WSSQ 12 items larger than 41 can be viewed as a high level of WRSS [41]. The Chinese version of the WSSQ had excellent internal consistency in the current study (Cronbach’s α = 0.93) and previous research (Cronbach’s α = 0.88) [40].

#### 2.2.3. Smartphone Addiction: Smartphone Application-Based Addiction Scale (SABAS)

SABAS items were scored along 6-point Likert-type scales, from 1 (strongly disagree) to 6 (strongly agree), and the scale assesses smartphone-addiction severity [42,43]. A sample item is “My smartphone is the most important thing in my life”. There are six self-reported items. A high level of SPA was defined when the participant scored 21 or above on the SABAS [44]. The Chinese version of SABAS has demonstrated good psychometric properties [42,43]. The internal consistency (Cronbach’s α) of the SABAS in the current study was 0.83, and in previous research, it was from 0.78 to 0.79 [43].

#### 2.2.4. Psychological Distress: Depression, Anxiety, Stress Scale-21 (DASS-21)

The Chinese version of DASS-21 was used to assess three types of negative affect (depression, anxiety, and stress) [45]. There are seven self-reported items in each subscale. Items were rated on a four-point Likert-type scale from 0 (“does not apply to me at all”) to 3 (“applies to me very much or most of the time”). Scores for each subscale were obtained by summation of all seven subscale items. Higher scores indicate greater corresponding negative affect in the past week. Moreover, a total score of the 21 DASS-21 items larger than 22 can be viewed as a moderate level of psychological distress [46]. The Cronbach’s α of the DASS-21 in the current study was 0.96, and in previous research, it was 0.95 [45].

#### 2.2.5. Demographics

In addition to the above scales, a demographic information sheet was used to understand participants’ backgrounds. Variables included age, gender, height, weight, marital status, disease, education level, and study major.

### 2.3. Statistical Analysis

Descriptive statistics were performed for the demographics of the entire sample and the moderate to high and low PA groups. In addition, t-tests and χ^2^ tests were used to examine differences in demographic variables between the two groups, and Cohens’ d was used to indicate the effect size. The variables analyzed in the present study could be viewed as normally distributed (skewness = 0.102 to 1.009; kurtosis = −0.580 to 0.532). Zero-order bivariate correlations were used to test relationships between every pair of variables, including age, weight status, psychological distress, SPA, and WRSS. For the moderated mediation model, Model 14 in the Hayes’ PROCESS macro and a bootstrapping method with 1000 bootstrapping resamples were used to conduct the analyses. More specifically, weight status (reference: non-overweight) was treated as the independent variable, SPA (reference: no) as the moderated variable, weight-related self-stigma as the mediated variable, and level of PA (reference: low) as the dependent variable. Furthermore, age and psychological distress were controlled in the model. All analyses were performed using SPSS 20.0 (SPSS Inc., Chicago, IL, USA).

## 3. Results

Table 1 displays demographics and studied variables for the entire sample (N = 391) and the groups with moderate to high (n = 127) and low (n = 264) PA. Briefly, the mean age was 22.85 years (standard deviation [SD] = 4.03), and mean BMI was 21.30 (SD = 3.53). There were significant differences on weight (*p* = 0.001), BMI (*p* < 0.001), weight status (*p* = 0.001), SPA (*p* < 0.001), and WRSS (*p* = 0.001) between the moderate to high and low PA groups. Table 2 displays the correlations between the studied variables. Except for age, all variables were positively correlated with each other (r = 0.14 to 0.45; *p*-values ranged from 0.007 to <0.001).

The results of Hayes’ PROCESS Model 14 showed that weight status was associated with WRSS (coefficient = 9.13, SE = 1.13, *p* < 0.001; 95% CI = 6.90, 11.36; see Table 3) and level of PA (odds ratio [OR] = 0.47, *p* = 0.03; 95% CI = 0.23, 0.93). WRSS (OR = 0.96, *p* = 0.03; 95% CI = 0.921, 0.997) and SPA (OR = 0.11, *p* = 0.003; 95% CI = 0.027, 0.468) were associated with level of PA. Moreover, SPA moderated the relationship between WRSS and level of PA (OR = 1.05, *p* = 0.049; 95% CI = 1.00, 1.10). However, the indirect effect was not significant in the presence of SPA (coefficient = 0.05, SE = 0.16; 95% CI = −0.24, 0.38). The overall moderated mediation model was not supported, with the index of moderated mediation = 0.44 (95% CI = −0.017, 0.960).

## 4. Discussion

The present study was conducted to assess relationships between WRSS, SPA, and level of PA in female college students. Specifically, a moderating effect of SPA on the association between WRSS and PA level was investigated. Both WRSS and SPA were related to the level of PA. When SPA was included as a moderator in a model to measure how this variable may impact the relation between WRSS and PA, a significant moderation effect was found, and WRSS was not found to mediate the relationship between weight status and PA. The implications are discussed below.

The association between WRSS and the level of PA has been observed previously among college students and young adults [29,30,47]. For example, Fung et al. [48], in a cross-sectional study, proposed a model to explain associations between the theory of planned behavior, WRSS, and PA [48]. They recruited 325 participants and used structural equation modeling to assess the models’ fitness. The study indicated that WRSS could explain engagement in PA and people with overweight and without WRSS had different attitudes towards and participation in PA. Furthermore, WRSS was recognized as an important determinant of PA. These findings are consistent with our results and together suggest that different strategies may be needed to promote PA in people with overweight/obesity and WRSS. Schmalz [47] assessed the predictability of weight stigma, body esteem, and BMI relative to PA [47]. He collected data from 76 young adults who were registered in a weight management intervention. Associations between stigma consciousness and perceived PA competence, with mediating effects of BMI and body esteem, were investigated using path modeling. He found that although weight stigma could statistically predict PA, only body esteem and not BMI was identified as a mediator. This study, unlike ours, assessed the willingness of participants to participate in PA. We measured the self-reported PA level as the outcome variable. Nevertheless, in both studies, the relationships between the level of PA/perceived competence to participate in PA and weight stigma were meaningful, indicating that weight stigma may be associated with both willingness to engage in PA and actual participation. Therefore, interventions that address the reduction of WRSS may influence both subjective and objective PA-related domains.

We found two studies from China that examined associations between SPA and PA in college students. In the first study, Shi et al. [36] attempted to find how SPA and PA may relate to irrational procrastination in 6294 university students [36]. They classified participants into two groups: active/inactive and addicted/non-addicted. Although in their study, the outcome measure was procrastination, they found that insufficient PA along with SPA had direct associations with procrastination, and a combination of these two variables may intensify the severity of the outcome measures. This finding is consistent with our results showing that PA may be affected by SPA, both directly and indirectly. In a second study, Yang et al. [37] examined the moderating effect of types of exercise on the relationship between PA and SPA among 650 students [37]. Similar to the present study’s findings, PA and SPA were significantly associated, and people with lower PA were more likely to exhibit SPA. They also found that types of exercise moderated relationships between PA and SPA. Specifically, individuals who engaged in more intense levels of PA were less likely to be involved in SPA than people who performed mild levels.

We found that SPA may operate as a moderator in the relationship between WRSS and PA. Specifically, SPA may exacerbate this association; that is, a combination of SPA and WRSS may have stronger effects on lower levels of PA than each one individually on PA. Therefore, we tentatively conclude that overweight/obese people who experience WRSS may be at relatively higher risk of physical inactivity than people who are lean/of normal weight or who do not experience WRSS, and SPA for people with WRSS may intensify their physical inactivity. Although causal relationships between these variables are unknown and we cannot declare which variables may lead to another, several implications still can be made. First, WRSS concerns should be monitored especially for those who are overweight/obese. Several programs have been designed to reduce WRSS [49], and we suggest that such programs be considered for female university students who are overweight or obese. Additionally, female university students who are at risk of SPA should be identified and helped to reduce their problematic use of smartphones. According to the observed associations, it may be possible to improve PA after reducing WRSS and SPA. However, future studies are needed to provide strong evidence on directionality in the observed associations between PA, WRSS, and SPA, for example, via the study design of longitudinal study or randomized controlled trials.

We did not find prior studies assessing the moderating effect of SPA on the association between WRSS and PA. Thus, we believe that this is the first study that does so. However, the current study has limitations. First, we used a cross-sectional design; thus, finding any causal relationship is impossible, and experimental studies are needed to further examine these associations. Second, we included only female college students in the data collection process. Therefore, we cannot generalize the findings beyond females, and the relationships between SPA, WRSS, and PA may be different in males. Third, we used an online survey to collect data that may be less accurate than face-to-face methods. However, this type of data collection may provide more accessibility for participants and does not convey a sense of being under supervision when completing the questionnaires. Finally, using self-report scales, especially for outcome measures of PA, may be vulnerable to recall bias. Nevertheless, the instrument used here (IPAQ) has been validated in many countries with different languages and cultures, mitigating this possibility.

## 5. Conclusions

This study shows that female university students with WRSS who are engaged in SPA may be at higher risk of low PA and that SPS may intensify the negative effects of WRSS on PA. Therefore, it is recommended that people, especially those with overweight/obesity and those more likely to experience WRSS, should be particularly careful regarding excessive use of smartphones, because this may decrease PA and expose them to further complications of sedentary lifestyles. Furthermore, healthcare providers should focus preventive strategies and treatment programs on young female students who feel WRSS, and are at risk of SPA, in order to keep them physically active.

## Figures and Tables

**Table 1:** Participants’ characteristics for the entirety (N = 391), and those with high (n = 48), moderate (n = 79), and low (n = 264) physical activity (PA).

Variable	n (%) or M (SD)				t or χ^2^ (*p*-Value)	Cohens’ d
	Entire Sample	High PA	Moderate PA	Low PA		
**Age (year)**	22.85 (4.03)	22.56 (3.38)	22.94 (3.47)	22.88 (4.29)	0.19 (0.85)	0.02
**Height (cm)**	160.82 (5.47)	161.50 (5.86)	161.60 (5.10)	160.46 (5.48)	1.87 (0.06)	0.20
**Weight (kg)**	55.08 (9.52)	55.70 (7.38)	51.25 (7.61)	56.12 (10.10)	3.42 (0.001)	0.35
**Body mass index (kg/m^2^)**	21.30 (3.53)	21.35 (2.63)	19.66 (3.01)	21.78 (3.68)	4.25 (<0.001)	0.44
**Marital status**					0.65 (0.42)	--
Single	367 (93.9)	46 (95.8)	75 (94.9)	246 (93.2)		
Others	24 (6.1)	2 (4.2)	4 (5.1)	18 (6.8)		
**Major**					5.38 (0.15)	--
Art and social science	242 (61.9)	34 (70.8)	44 (55.7)	164 (62.1)		
Science and engineering	57 (14.6)	6 (12.5)	14 (17.7)	37 (14.0)		
Medicine and biology	54 (13.8)	4 (8.3)	8 (10.1)	42 (15.9)		
Others	38 (9.7)	4 (8.3)	13 (16.5)	21 (8.0)		
**Weight status**					10.17 (0.001)	--
Overweight/obese	76 (19.4)	8 (16.7)	5 (6.3)	63 (23.9)		
Lean/non-overweight	315 (80.6)	40 (83.3)	74 (93.7)	201 (76.1)		
**Moderate to high PA (no)**	264 (67.5)	--	--	--	--	--
**Disease (no)**					2.93 (0.09)	--
No	366 (93.6)	43 (89.6)	72 (91.1)	251 (95.1)		
Yes	25 (6.4)	5 (10.4)	7 (8.9)	13 (4.9)		
**Smartphone addiction**					19.80 (<0.001)	--
No	145 (37.1)	21 (43.7)	46 (58.2)	78 (29.5)		
Yes	246 (62.9)	27 (56.3)	33 (41.8)	186 (70.5)		
**Weight-related self-stigma**	30.49 (10.58)/12–60	30.40 (10.01)	26.37 (10.82)	31.74 (10.33)	3.42 (0.001)	0.37
**Psychological distress**	13.39 (12.61)/0–63	13.62 (12.12)	11.32 (13.56)	13.97 (12.39)	1.31 (0.19)	0.14

**Note:** *t*-tests, χ^2^ tests, and Cohen’s d were calculated for comparing low PA group with moderate to high PA group.

**Table 2:** Zero-order bivariate correlation coefficients between studied variables in female university students.

			r (*p*-Value)		
	1.	2.	3.	4.	5.
1. Age	--				
2. Weight status	−0.01 (0.78)	--			
3. Psychological distress	0.03 (0.55)	0.14 (0.007)	--		
4. Smartphone addiction	−0.02 (0.67)	0.18 (<0.001)	0.33 (<0.001)	--	
5. Weight-related self-stigma	0.04 (0.40)	0.40 (<0.001)	0.45 (<0.001)	0.42 (<0.001)	--

**Table 3:** Multivariate regression models explaining moderate to high physical activity (PA) among female university students (N = 391).

	Weight-Related Self-Stigma		Moderate to High PA	
	Coeff. (SE)/*p*-Value	LLCI, ULCI	OR (*p*-Value)	LLCI, ULCI
Age	0.09 (0.11)/0.40	−0.12, 0.31	0.99 (0.68)	0.93, 1.05
Weight status (Ref: non-overweight group)	9.13 (1.13)/<0.001	6.90, 11.36	0.47 (0.03)	0.23, 0.93
Psychological distress	0.34 (0.04)/<0.001	0.27, 0.41	1.01 (0.39)	0.99, 1.03
Smartphone addiction (Ref: no)	--	--	0.11 (0.003)	0.03, 0.47
Weight-related self-stigma	--	--	0.96 (0.03)	0.922, 0.997
Weight-related self-stigma × Smartphone addiction	--	--	1.05 (0.049)	1.0001, 1.1004
	**F-Value (*p*-Value)**	**R^2^**	**−2LL (*p*-Value)**	**Cox & Snell’s R^2^**
	59.71 (<0.001)	0.32	461.12 (<0.001)	0.078

−2LL = −2 log likelihood statistics; OR = odds ratio; LLCI = lower limit of confidence interval at 95%; ULCI = upper limit of confidence interval at 95%; SE = standard error; Coeff. = coefficient.

## Data Availability

The data presented in this study are available on request from the corresponding author. The data are not publicly available due to the regulation in the Institutional Review Board.
